# Stanniocalcin-1 Reduces Tumor Size in Human Hepatocellular Carcinoma

**DOI:** 10.1371/journal.pone.0139977

**Published:** 2015-10-15

**Authors:** Bonnie H. Y. Yeung, Felix H. Shek, Nikki P. Lee, Chris K. C. Wong

**Affiliations:** 1 Department of Biology, Hong Kong Baptist University, Kowloon Tong, Hong Kong; 2 Department of Surgery, The University of Hong Kong, Pokfulam, Hong Kong; University of Medicine, Greifswald, Germany, GERMANY

## Abstract

Growing evidence has revealed high expression levels of stanniocalcin-1 (STC1) in different types of human cancers. Numerous experimental studies using cancer cell lines demonstrated the involvement of STC1 in inflammatory and apoptotic processes; however the role of STC1 in carcinogenesis remains elusive. Hepatocellular carcinoma (HCC) an exemplified model of inflammation-related cancer, represents a paradigm of studying the association between STC1 and tumor development. Therefore, we conducted a statistical analysis on the expression levels of STC1 using clinicopathological data from 216 HCC patients. We found that STC1 was upregulated in the tumor tissues and its expression levels was positively correlated with the levels of interleukin (IL)-6 and IL-8. Intriguingly tumors with greater expression levels of STC1 (tumor/normal ≥ 2) were significantly smaller than the lower level (tumor/normal<2) samples (p = 0.008). A pharmacological approach was implemented to reveal the functional correlation between STC1 and the ILs in the HCC cell-lines. IL-6 and IL-8 treatment of Hep3B cells induced STC1 expression. Lentiviral-based STC1 overexpression in Hep3B and MHCC-97L cells however showed inhibitory action on the pro-migratory effects of IL-6 and IL-8 and reduced size of tumor spheroids. The inhibitory effect of STC1 on tumor growth was confirmed *in vivo* using the stable STC1-overexpressing 97L cells on a mouse xenograft model. Genetic analysis of the xenografts derived from the STC1-overexpressing 97L cells, showed upregulation of the pro-apoptotic genes interleukin-12 and NOD-like receptor family, pyrin domain-containing 3. Collectively, the anti-inflammatory and pro-apoptotic functions of STC1 were suggested to relate its inhibitory effect on the growth of HCC cells. This study supports the notion that STC1 may be a potential therapeutic target for inflammatory tumors in HCC patients.

## Introduction

Stanniocalcin-1 (STC1) is a hypocalcemic hormone, synthesized and secreted by a unique endocrine gland, corpuscles of Stannius (CS) in bony fish. There is no CS gland or any comparable structure found in mammals, the STC1 gene was believed to have been lost in evolution. The mammalian forms of STC1, however, were cloned in gene-screening experiments using differential mRNA expression in mouse cDNA and human expressed sequence tag samples [1–3], and was then found to be broadly expressed in various body tissues [4]. Early studies intended to mirror the fish data and to validate the endocrine effects of STC1 on Ca^2+^ homeostasis in mammals. However many studies illustrated the mammalian STC1 exerts its functions via paracrine/autocrine pathways [5], which is different from the action reported in fish models. In addition, a study using STC1 (-/-) null mice revealed that STC1 is not much involved in blood Ca^2+^-regulation in mammals [6]. Unexpectedly, considerable studies reported the involvement of the mammalian STC1 in processes of inflammation and carcinogenesis [7].

In clinicopathological study of STC1 functions, significant elevated STC1 expression levels were mostly detected in different human cancer samples, such as tumors of lung, breast, ovary, colon, pancreas, and liver [7]. The elevated expression levels of STC1 identified in patients with different cancer types was found to correlate to poor prognosis; hence, the use of STC1 as a molecular marker for cancer progression has been suggested [7, 8]. In experimental studies of analyzing STC1 functions, the use of nude mice xenograft models to study growth and metastasis of different tumor cells, however has produced inconclusive results. Experimental studies using ovarian [9], gastric [10], and colorectal [11] tumor cells in nude mice supported the pro-oncogenic role of STC1. In another study, an anti-oncogenic role of STC1 via inhibiting proliferation and invasion of cervical cancer cells was reported [12]. Accompanied with clinical analysis and nude mice studies, experimental investigations using human cancer cell lines identified various transcriptional factors that regulated STC1 expression in the process of tumor progression. For example, hypoxia-inducible factor (HIF)-1α [13, 14], p53 [15], Sp1 [16], RET-multiple endocrine neoplasia type 2B mutant protein [17], BRCA1 [18], and vascular endothelial growth factor (VEGF) [19–23] were shown to stimulate STC1 expression. Although the underlying mechanistic actions of STC1 on tumor progression are not immediately obvious, a considerable number of experimental studies using different cancer cell lines demonstrated that STC1 was involved in Warburg effect, apoptosis, angiogenesis, and wound healing [14, 24–26]. Mitochondrial proteins (e.g., uncoupling factor 2) [27] and/or IL-6 [28] were reported to be involved in the regulation or function of STC1, suggesting that STC1 may participate in the modulation of mitochondrial antioxidant functions and cellular/tissue inflammatory responses [29]. Despite the current evidence shows an association between STC1 and cancer progression, further investigation is necessary to reveal at the mechanistic perspectives of its actions and effects on carcinogenesis.

Hepatocellular carcinoma (HCC) is the seventh most common cancer worldwide and the third most common cause of death from cancer worldwide. HCC development is mainly due to chronic inflammation and is therefore considered as an inflammatory cancer [30]. The chronic inflammation is characterized by the continued expression of cytokines and recruitment of immune cells to the liver, where the interaction with tumor cells is recognized to be crucial for HCC development [31]. STC1 was found to be involved in inflammatory events [28, 29, 32] and was expressed in hepatocarcinomatous tissues [33, 34]; however, the role of STC1 in HCC progression is not known. In this study, clinicopathological and microarray profiling data of paired tumor and adjacent normal tissues from 216 HCC patients were analyzed. Experimental studies using various HCC cell-lines and nude mice xenograft model were conducted to reveal the roles of STC1 in HCC progression. Our data suggested that STC1 inhibited pro-migratory effects of IL6/IL8 and enable apoptotic pathways in tumor cells to reduce growth and metastasis of HCC.

## Materials and Methods

### Patient cohorts and clinical samples for microarray analysis

All patients enrolled in this study underwent a curative hepatectomy for HCC at Queen Mary Hospital, Pokfulam, Hong Kong, between 1993 and 2007. The Institutional Review Board for Human Ethics, The University of Hong Kong/Hospital Authority Hong Kong West Cluster approved the study on the collection of clinical specimen for research. Written informed consent was obtained from patients regarding the use of the liver specimens for research. The participant consent forms were filed in the tissue bank in the Department of Surgery, The University of Hong Kong. The samples were screened by microarray analysis as described previously [35]. Statistical analyses were conducted by GraphPad Prism 6 (GraphPad Software Inc., La Jolla, CA) or SPSS 16.0 for Windows (SPSS, Chicago, IL). Pearson’s chi-squared test and Student’s *t*-test were used for calculating the *p*-value, which was defined as statistically significant at P < 0.05.

### RNA, protein extraction, western blotting and quantitative PCR

Total RNA from cell lines, frozen patient samples, and xenografts was extracted using TRIzol reagent according to the manufacturer’s instruction (Life Technologies, Carlsbad, CA). Five hundred nanograms of patient RNA was reverse transcribed to cDNA using TaqMan Reverse Transcription Kits (Applied Biosystems, Foster City, CA) while RNA of cell lines was reverse transcribed using HC RNA-cDNA Master Mix (Applied Biosystems). Quantitative PCR (qPCR) was performed using Fast SYBR Green Master Mix (Applied Biosystems). The primer sequences for STC1, IL-6, IL-8, IL-12A, IL-12B, NLRP3 and β-actin were as follows: *STC1*: 5’-TGAGGCGGAGCAGAATGACT-3’ and 5’-CAGGTGGAGTTTTCCAGGCAT-3’; *IL6*: 5’-AGCCCACCGGGAACGAAAGA-3’ and 5’-TGTGTGGGGCGGCTAC ATCT-3’; *IL8*: 5’-AAGCCACCGGAGCACTCCAT-3’ and 5’-CACGGCCAGCTTG GAAGTCA-3’; *IL12A*: 5’-GAATGCAAAGCTTCTGATGGA-3’ and 5’-tcaaggga ggatttttgtgg-3’; *IL12B*: 5’-ccctgacattctgcgttca-3’ and 5’–aggtc ttgtccgtgaagactcta-3’; *NLRP3*: 5’-TGAAGAGGAGTGGATGGGTT-3’ and 5’-GTCGTGTGTAGCGTTTGTTG-3’ and *β-actin*: 5’-GACTACCTCATGAAGA TCCTCACC-3’ and 5’-TCTCCTTAATGTCACGCACGATT-3’. The thermocycling protocol was, 95°C for 1 min, followed by 40 cycles of 95°C for 10 s, 56°C for 10 s and 72°C for 30 s using the ABI StepOne Real-time PCR System (Applied Biosystems).

For western blotting, protein lysates were prepared using RIPA buffer (50 mM Tris-HCl, pH 7.4, 150 mM NaCl, 2mM EDTA, 1% NP-40, 0.1% SDS), supplemented with complete protease and PhosSTOP Phosphatase Inhibitor Cocktail Tablets (Roche Life Sciences, Mannheim, Germany). Protein concentrations were determined using the DC Protein Assay Kit II (Bio-Rad, Hercules, CA). Proteins lysates were resolved by SDS-PAGE gels and were blotted as described previously [13]. Blotting was conducted using mouse antibody against V5 (Invitrogen, Carlsbad, CA), rabbit antibodies against human STC1 (Origene, Rockville, MD), p-ERK1/2, total-ERK1/2 (both from Cell Signaling Technology, Danvers, MA), anti-actin (Sigma-Aldrich, St. Louis, MO), goat antibodies against human IL-6 or human IL-8 (R&D Systems, Minneapolis, MN), followed by an incubation with horseradish peroxidase (HRP)-conjugated goat anti-mouse/rabbit antibody (1:4000, BioRad, Hercules, CA, USA). Protein bands were visualized using the chemiluminescent reagent (WestSave Up, AbFrontier, Seoul, South Korea).

### Cell culture

Immortalized human hepatocyte MIHA, primary HCC (HepG2, Hep3B, HuH7, and H2P), and metastatic HCC (MHCC-97L, MHCC-97H, H2M) cell lines were cultured in high-glucose Dulbecco’s modified Eagle’s media (Life Technologies) supplemented with antibiotics (50 U/mL penicillin and 50 μg/mL streptomycin) (Life Technologies), 10% heat-inactivated fetal bovine serum (FBS) (HyClone; Life Technologies) and seeded overnight before treatments. The human embryonic kidney cell line HEK293FT (Life Technologies) was cultured in the same medium, supplemented with 6mM L-glutamine (Sigma-Aldrich) and 1 mM sodium pyruvate (Sigma-Aldrich). Cells were serum starved overnight before an addition of recombinant IL-6 and IL-8 (R&D Systems).

The basal levels of STC1 protein in cell lysates and conditioned cell culture media were measured by an enzyme-linked immunosorbent assay (ELISA), using the DuoSet ELISA kit (R&D Systems), according to the manufacturer’s instruction. Briefly, wells of microplates were coated with goat anti-human STC1 antibody at room temperature overnight, followed by blocking with the reagent diluent (phosphate-buffered saline (PBS) containing 1% bovine serum albumin). Either samples or diluted STC1 standard solutions (62.5–4000 pg/mL) were added into the antibody-coated wells, followed by an incubation with the biotinylated goat anti-human STC1 antibody and streptavidin-HRP. After the addition of the substrates H_2_O_2_ and tetramethylbenzidine (TMB) (R&D Systems), the reaction was stopped using one molar H_2_SO_4_. The absorbance at 450 nm was determined using an ELx800 microplate reader (BioTek, Winooski, VT).

### Overexpression of STC1

#### Construction of pLenti6.3/TO/V5-DEST-STC1 plasmid

Human *STC1* cDNA encoding the wild-type full-length protein without the stop codon was amplified by PCR and cloned into pENTR/SD/D-TOPO (Life Technologies) according to the manufacturer’s instruction. The STC1 insert was then cut from pENTR/SD/D-TOPO and cloned into the expression vector pLenti6.3/TO/V5/-DEST (Life Technologies) using Gateway LR Clonase II Plus Enzyme Mix (Life Technologies).

#### Production of lentivirus in 293FT and lentiviral overexpression of STC1 in Hep3B or MHCC-97L cells

Overexpression of STC1 was established using a ViraPower Lentiviral Expression System (Life Technologies) and conducted as described in our previous study [25]. Briefly, HEK293FT cells were seeded into 100-mm dishes overnight, then cotransfected with ViraPower Packaging Mix and pLenti6.3/TO/V5-DEST-STC1 or pLenti6.3/TO/V5-DEST using Lipofectamine 2000. Viral supernatants were harvested at 48 h after the transfection and were immediately used or stored at −80°C. Hep3B or MHCC-97L cells were seeded at 1 × 10^5^ cells/well into 6-well plates overnight, and then transduced with 1mL of lentiviral particles with 6 μg/mL of polybrene (Sigma-Aldrich) for 24 h. Stable cell lines were selected under 4 μg/mL blasticidin (Life Technologies) treatment over 2 weeks. Overexpression of STC1 in Hep3B cells (Hep3B/STC1) or MHCC-97L cells (97L/STC1) was established in parallel with pLenti6.3/TO/V5-DEST overexpression in Hep3B (Hep3B/pLenti) or in MHCC-97L (97L/pLenti). The expression level of STC1 was confirmed by qPCR and western blotting.

### Spheroid formation assay

A spheroid formation assay was performed using Costar 24-well clear, flat-bottom, ultra-low attachment microplates (Corning, Corning, NY). Cell suspensions (2 × 10^3^/well) were dispensed into the plate with serum-free culture medium supplemented with 20ng/mL h-EGF (Sigma-Aldrich), h-IGF and hFGF (both from Cell Signaling Technologies). Every 2–3 days, 200 μL of fresh supplements were added into each well. After 10 days of incubation, tumor spheroids were imaged and measured under a microscope at 200× magnification (Motic Asia, Hong Kong).

### Boyden chamber-based cell migration assay

Migration assays were performed using 24-well Transwell inserts with 8-μm pore size membranes (Costar, Corning). Stably transduced Hep3B cells were trypsinized and washed twice with serum-free medium. Then, the cells (6 × 10^4^) were seeded into inserts (upper chamber) with or without interleukins, while the complete medium was added at the lower chamber as chemoattractant. After 24-h incubation at 37°C, the cells on the top of the insert membrane were removed by cotton swabs and the cells at the bottom (migrated cells) were rinsed with PBS, fixed with methanol for 10 min at −20°C, and stained with 0.5% Crystal Violet (Farco Chemical Supplies). The total number of migrated cells was counted using microscopy and cell morphology was captured at 100× magnification.

### Measurement of secreted IL-6 and IL-8 by ELISA

The secretion of IL-6 and IL-8 was detected by the human ELISA kits, according to the manufacturer’s instruction (ExCell, Shanghai, China). Briefly, 100 μL of standards, samples, or diluents were added into each well and incubated for 90 min at 37°C. Then the wells were rinsed with wash buffer 4 times, followed by an addition of 100 μL diluted biotinylated antibodies to the wells (except the blank) and incubated for 60 min at 37°C. The wells were washed and 100 μL of diluted HRP-conjugated secondary antibody was added to the wells (except the blank) and incubated for 30 min at 37°C. After the washing step, 100 μL of TMB substrate was added into the wells and incubated for 15 min at 37°C. Following the incubation, 100 μL of Stop Solution was added into the wells and absorbance was measured at 450 nm against the background signal at 690 nm using the ELx800plate reader.

### Xenograft animal tumor model

Animal experiments were approved by the Animals (Control of Experiments) Ordinance 340, Hong Kong, and were conducted according to the guidelines of the Committee on the Use of Live Animals in Teaching and Research (CULATR), The University of Hong Kong, Hong Kong (Ref: 2825–12). Two hundred microliters of 97L/pLenti or 97L/STC1 cells (3 × 10^6^) were inoculated subcutaneously in the right flank of 6–7 week-old male mice (BALB-c nude) (n = 10) using 29-gauge needles. Tumor volumes were measured every week and calculated by a standard equation [(width^2^×length)/2]. When the mean tumor volume of 97L/pLenti-inoculated mice reached approximately 700 mm^3^, all mice were sacrificed by anaesthetizing with pentobarbital followed by cervical dislocation. The xenograft tumors in nude mice was dissected and examined using hematoxylin-eosin staining. Levels of STC1 mRNA were measured using real-time PCR (data not shown). Data are shown as means ± SEM. A *p* value of <0.05 was considered statistically significant. All studies were performed in duplicate for two independent sets of the experiments.

### Statistical analysis

All *in vitro* assays were analyzed by Student’s *t*-test or one-way analysis of variance (ANOVA) followed by Duncan’s multiple range test. All data are shown as means ± SD. A *p-*value of < 0.05 was considered statistically significant. All studies were performed in duplicate for three independent sets of experiments.

## Results

**Fig 1:**
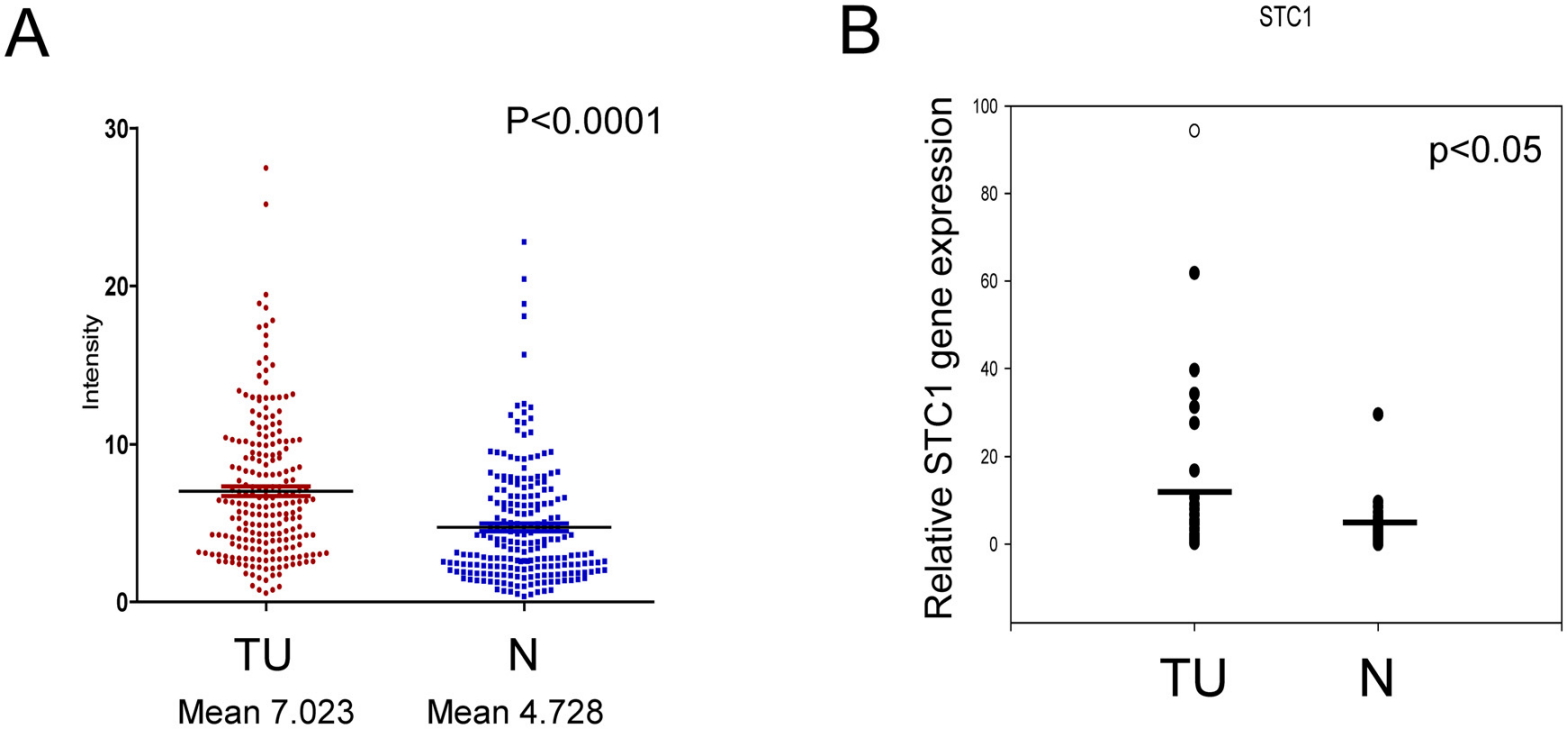
STC1 was upregulated in HCC tumor tissues. *STC1* gene expression level was detected and measured in both tumor (TU) and normal (N) patient samples by using microarray analysis (A) and qPCR (B). *STC1* gene expression was significantly greater in the TU samples vs normal tissues.

Using a cohort of patients with HCC (n = 216), the *STC1* gene expression level was examined in paired HCC normal (N) and tumor (TU) tissues by microarray. To describe the high and normal expression levels of *STC1* in the patient samples, a ratio of TU/N ≥ 2 was defined to denote high expression (STC1-High) whereas a ratio < 2 indicated low or normal expression (STC1-Normal). Using these criteria, 40% of the cases were classified as STC1-High (85 cases out of 216), and 60% of the cases were STC1-Normal (131 cases out of 216) (Table 1). Tumor size of the cohort with STC1-High (mean 6.359) versus STC1-Normal (mean 7.841) was significantly reduced (*p* = 0.008), which was not associated with sex, age, tumor size with 5-cm cutoff, alpha fetoprotein level, hepatitis B surface-antigen level, metastasis stage, histological differentiation, infiltration, recurrence, and cirrhosis (Table 1). Indeed, the cohort with STC1-High was not correlated with prolonged survival (S1 Fig). Expression of *STC1* was determined in tumor-normal tissue paired samples using both microarray and qPCR. The microarray data showed that the expression of *STC1* mRNA was increased by 1.49-fold in tumor tissues (mean 7.023) as compared with their normal counterparts (mean 4.728) (*p*<0.0001, Fig 1A). To confirm the gene expression data from microarray, qPCR was performed using 38 tumor-normal paired samples. Consistent with the microarray data, the expression levels of *STC1* in tumor tissues was greater than the corresponding normal tissues (2.89-foldincrease, *p*<0.05; Fig 1B). We then attempted to establish a correlation between STC1 with two well-characterized cytokines, IL-6 and IL-8, among HCC cases. In the analysis of the clinicopathological data, STC1 expression level was found to be positively associated with IL-6 (*p*<0.0001) and IL-8 (*p* = 0.0004) in HCC tumors using Pearson’s correlation analysis (Table 2).

**Table 1:** The comparison of STC1 expression levels with clinicopathological data in HCC patients. HCC cases (n = 216) was divided into 2 cohorts, STC1 high (STC1-High, TU/N ≥2) and STC1 low or normal group (STC1-Normal, TN/N <2). Tumors with STC1-High were found to associate with smaller size tumors. Chi-squared test was used to calculate the significance among different parameters. p<0.05 was considered as significant (n = 216).

Variables	Frequency (%)	STC1-Normal	STC1-High	P-value
***Sex***				
Male	170 (78.7)	102	68	0.737
Female	46 (21.3)	29	17	
***Age***				
<60	131 (60.6)	79	52	1
≥60	85 (39.4)	52	33	
***Tumor size (cm)***				
<5	87 (40.3)	47	40	0.119
≥5	129 (59.7)	84	45	
***Alpha fetoprotein (ng/mL)***				
<250	126 (58.3)	76	50	1
≥250	90 (41.7)	55	35	
***Hepatitis B surface antigen*** *				
Negative	30 (14)	21	9	0.317
Positive	185 (86)	110	75	
***Pathological tumor-node-metastasis stage***				
Early (I, II)	101 (46.8)	60	41	0.781
Late (III, IV)	115 (53.2)	71	44	
***Histological differentiation*** *				
Well	37 (19.8)	22	15	0.71
Moderate/Poor	150 (80.2)	94	56	
***Venous infiltration***				
Absent	109 (50.5)	66	43	1
Present	107 (49.5)	65	42	
***Recurrence***				
Absent	99 (45.8)	57	42	0.406
Present	117 (54.2)	74	43	
***Cirrhosis*** *				
Negative	208 (96.7)	127	81	0.438
Positive	7 (3.3)	3	4	
***Tumor size (cm)***				
Mean		7.841	6.359	**0.008**
SD		4.32	3.75	
***Number of tumor nodules***				
Mean		2.27	2.16	0.77
SD		2.77	2.65	

*Incomplete patient information.

**Table 2:** Expression level of *STC1* was correlated with *IL6* and *IL8* in HCC tumors (n = 220) using Pearson’s correlation analysis.

	STC1	
Parameter	IL6-TU	IL8-TU
Number of XY Pairs	220	220
Pearson r	0.2937	0.2352
95% confidence interval	0.1679 to 0.4101	0.1062 to 0.3564
P-value (two-tailed)	<0.0001	0.0004
P-value summary	***	***
Is the correlation significant? (alpha = 0.05)	Yes	Yes
R square	0.08625	0.05531

**Fig 2:**
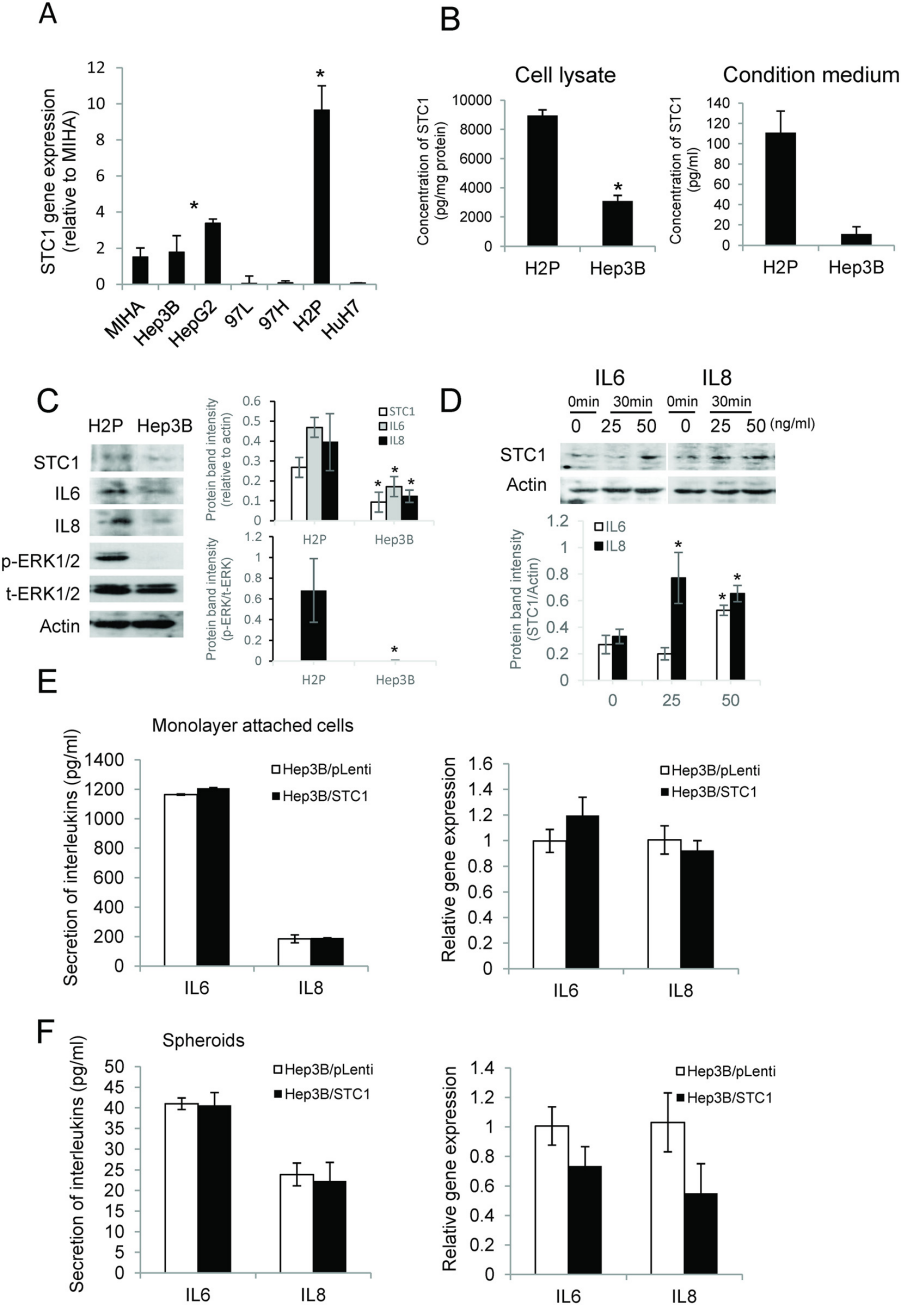
STC1 was a downstream target of IL6 and IL8. (A) *STC1* gene expression was determined in a panel of HCC cell lines using qPCR. (B) STC1 protein levels in cell lysates and condition media of H2P and Hep3B cells were measured using ELISA. (C) Western blotting analysis of STC1, IL6, IL8 and ERK1/2 in H2P and Hep3B cell lines. (D) After overnight serum starvation, addition of 25 and 50 ng/ml of IL6 or IL8 in Hep3B cells for 30 min induced STC1 protein expression. The secretion (*left*) and gene expression (*right*) of both IL6 and IL8 were measured in Hep3B monolayer attached cells (E) and 10 days’ spheroids (F).

To characterize the role of STC1 in hepatocarcinogenesis, expression levels of STC1 in different human HCC cell lines were determined and *in vitro* functional assays were then conducted. The data revealed that low STC1 expression levels was detected in most of the HCC cell lines, but the expression level was greater in H2P cells (Fig 2A). Consistently, the data of ELISA showed significant high levels of STC1 protein detected in cell lysates and condition media of H2P cells (Fig 2B). To compare the expression of profiles of STC1, IL6, IL8 and pERK, protein lysates were prepared from Hep3B and H2P cells. H2P cells showed greater expression levels of IL-6, IL-8 and p-ERK1/2 (137F5) (Fig 2C). The western blot data of IL6 and IL8 support the correlation analysis of the clinicopathological data from the patient samples (Table 2) and the findings reported by Westerberg et al. [28], showing STC1 expression was induced by IL-6 via MAPK signaling. To investigate the possible cause-and-effect relationship between STC1 and the cytokines IL6 and IL8, a pharmacological approach was adopted to elucidate the possible interaction between IL-6, IL-8 and STC1 using Hep3B cells. Upon stimulation by 50 ng/mL of IL-6 or IL-8 for 30 min, STC1 protein expression was found to be upregulated (Fig 2D). On the contrary, to address the effects of STC1 on IL6/IL8 expression, STC1-overexpressed Hep3B cells (Hep3B/STC1) and the empty vector control Hep3B/pLenti were generated using lentiviral system. The overexpression of STC1 was verified using qPCR (data not shown) and western blotting (Figure A in S2 File). The STC1-overexpressing cells, either cultured in monolayers (Fig 2E) or spheroids (Fig 2F) condition, did not show noticeable effects on the secretion (*left*) and mRNA expression (*right*) of IL-6 or IL-8. Collectively, the data suggest that STC1 is a downstream target of IL6/IL8 in Hep3B cells.

**Fig 3:**
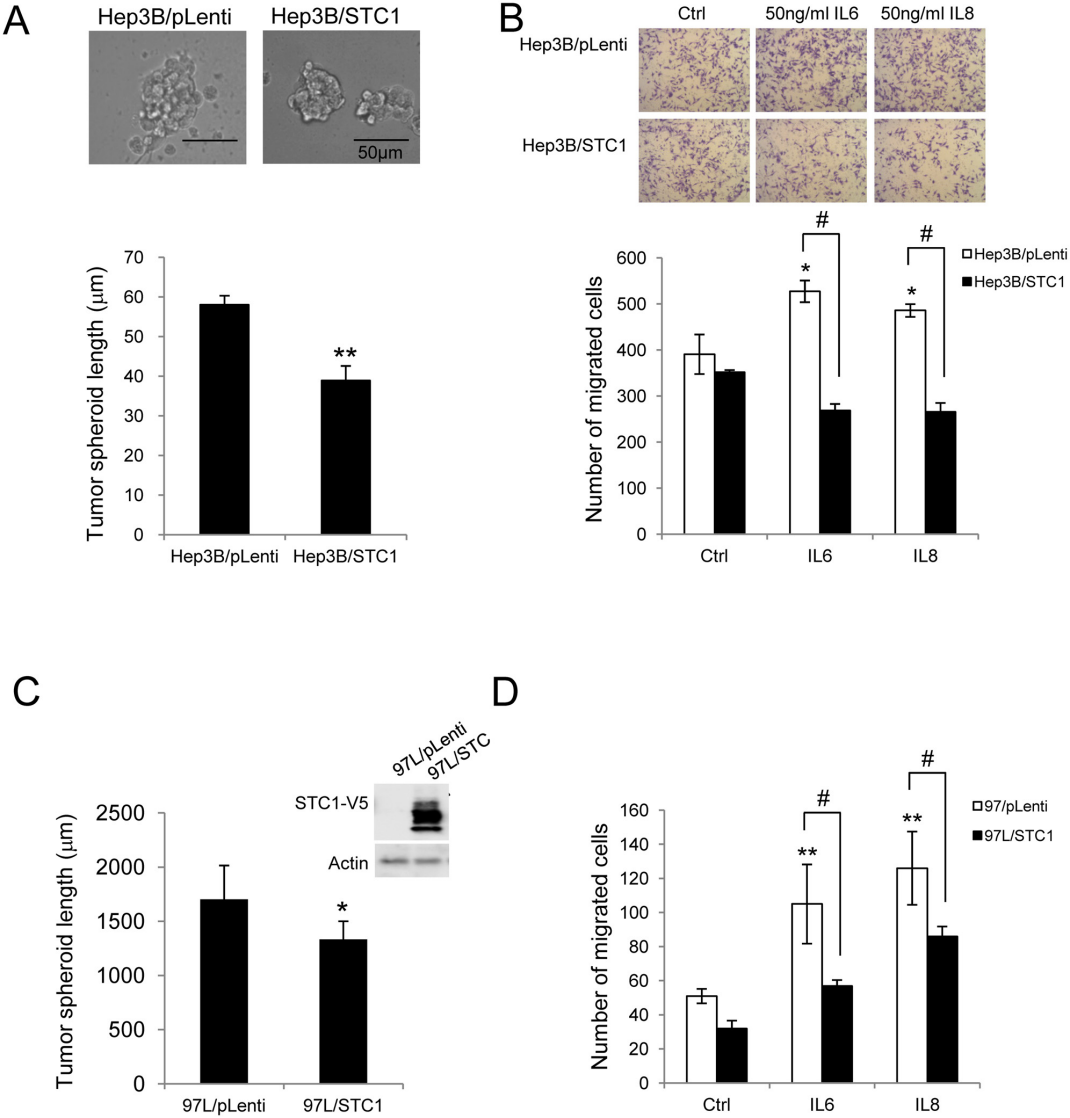
STC1 suppressed HCC tumor growth *in vitro*. Spheroid formation and cell migration assay were performed in both stably STC1-overexpressed Hep3B (A-B) and 97L cell lines (C-D). The sizes of spheroids were measured at 10 days of the incubation. At least 3 random fields representing 200 spheroids were counted under 200x magnification. (A) In Hep3B cells, the representative pictures of spheroids were shown (*top*). (C) In 97L cells, overexpression of STC1 was confirmed by probing with V5 antibody (*right*). Significant reductions in sizes of the spheroids in Hep3B/STC1 and 97L/STC1 were noted. * p<0.05, **p<0.01, compared with empty vector transduced cells. Bars = 50 μm. The activities of cell migration in transwells were induced in (B) Hep3B cells and (D) 97L cells by the treatment with 50ng/ml of IL6 or IL8 for 24h. At least 5 random fields were counted under 100x magnification. The pro-migratory effects of IL6 or IL8 on Hep3B/STC1 and 97L/STC1 were significantly suppressed by STC1-overexpression. * p<0.05, ** p<0.001, compared with the treatments in the respective pLenti-transduced cells; # p<0.00001, compared with the same treatment group.

To characterize the observation of the STC1-High cohort with shrinkage of tumor size, an *in vitro* spheroid formation assay was implemented. Consistent with the clinicopathological data, the size of the tumor spheroids of Hep3B/STC1 cells cultured for 10 days, was significantly smaller (1.53-fold, *p*<0.01) than the spheroids of Hep3B/pLenti (Fig 3A). The viability of Hep3B/STC1 cells was significantly reduced after 3 days of the incubation, as determined by MTT proliferation assay (Figure B in S2 File). Since the expression of STC1 was positively correlated to the levels of IL6 and IL8 in HCC tumors, whereas IL-6 and IL-8 is known to exert pro-carcinogenic effects in HCC [36], the effect of STC1 on IL-6- or IL-8-elicited cell migration was investigated using HCC cell-lines. In the control cells (Hep3B/pLenti), both IL-6 or IL-8 enhanced cell migration by 1.35- or 1.83-fold, respectively, versus the vehicle treatment (*p*<0.05, Fig 3B). While the percentage of migration in STC1-overexpressed cells (Hep3B/STC1) was similar to the Hep3B/pLenti cells. However the pro-migratory effects of IL-6 or IL-8 were suppressed in the Hep3B/STC1 cells by 1.96- or 1.83-fold, respectively (*p*<0.0001, Fig 3B). To confirm the finding, another HCC cell line MHCC-97L (97L) with low basal STC1 expression was used. Stable overexpression of STC1 in 97L cells versus empty vector transduction was verified using qPCR (data not shown) and western blotting (Fig 3C, *right*). In 97L/STC1 cells, the size of the tumor spheroids, after 10 days of incubation was 1.28-fold smaller than the 97L/pLenti (*p*<0.05) (Fig 3C). Cell migratory activity was stimulated by IL-6 or IL-8 in the control cells (97L/pLenti) by 2.06- or 2.47-fold, respectively (*p*<0.001, Fig 3D), while the pro-migratory effects of IL6 or IL8 were suppressed by STC1-overexpression in 97L/STC1 by 1.84- or 1.47-fold, respectively (*p*<0.0001, Fig 3D).

**Fig 4:**
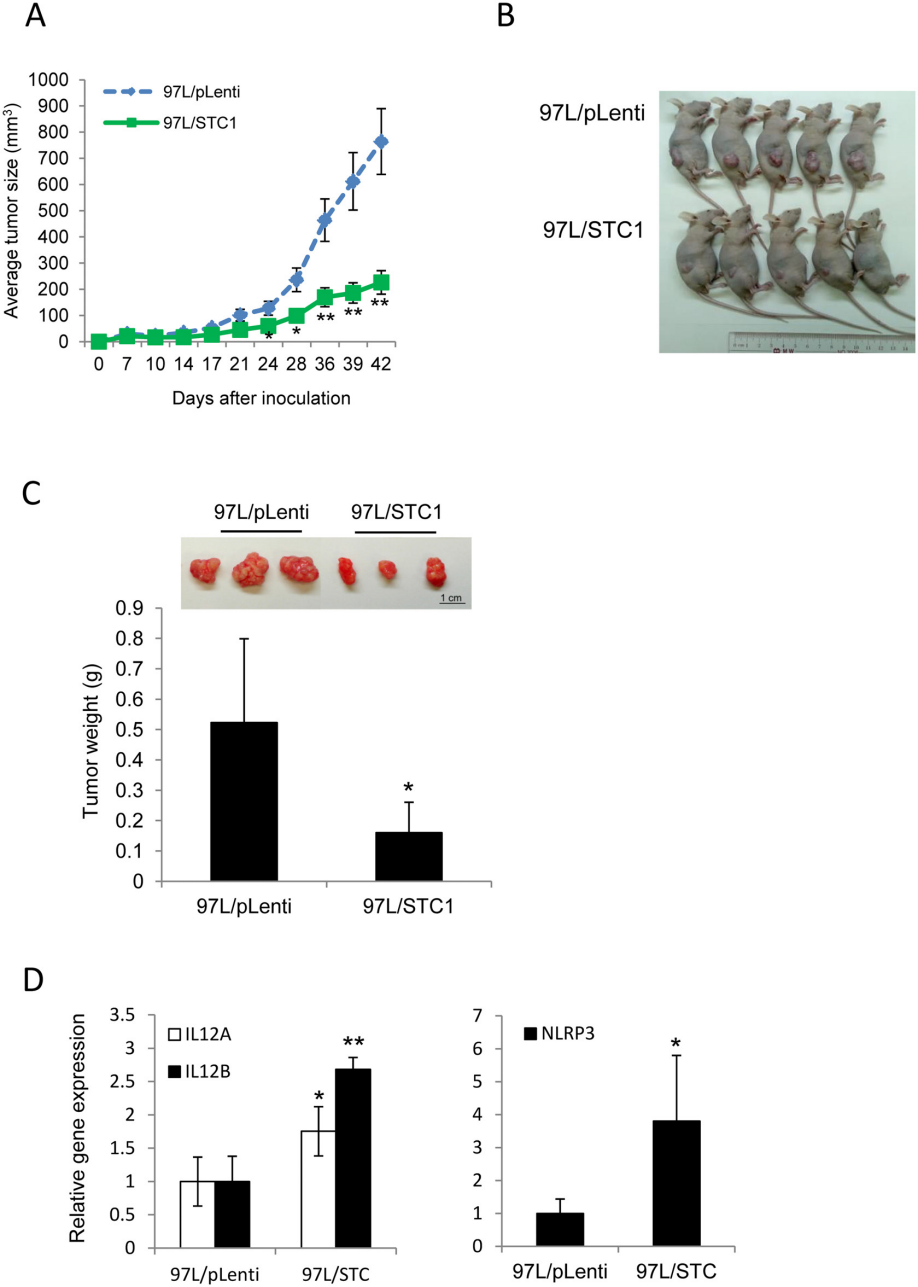
STC1 suppressed HCC tumor growth *in vivo*. The transduced MHCC-97L cells [pLenti (97L/pLenti) or STC1 (97L/STC1)] were inoculated in nude mice subcutaneously. (A) Tumor sizes were measured from 4 weeks of the inoculation (mean ± SEM). * *p*<0.05 and ** *p*<0.01, as compared with the 97L/pLenti group. (B) The mice were scarified (10 mice per group) on 47 days after the inoculation and representative mice were shown. (C) Tumor xenografts were dissected and weighted (mean ± SD), and the representative tumor masses were shown on *top*. Bar = 1cm. *, *p*<0.01, compared with the 97L/pLenti group. (D) The expression levels of selected pro-apoptotic genes in xenograft samples were measured using qPCR. * *p*<0.05 and ** *p*<0.0001, compared with the 97L/pLenti group.

To validate the in vitro data, nude mice xenograft model was used. We firstly attempted to inoculate Hep3B/STC1 or Hep3B/pLenti cells into nude mice, but no tumor growth was observed with either cell line (data not shown). Therefore 97L cell-line was used for the inoculation as it was successfully established in our previous study [37]. 97L/STC1 or 97L/pLenti cells were subcutaneously inoculated into nude mice (n = 10), and the animals were then monitored every 3 days. Tumor sizes were measured weekly starting from 4 weeks of the inoculation. Of the 10 mice injected with 97L cells, 100% developed tumors within 5 weeks of the injection. Starting from 24 days of the inoculation, the size of xenograft tumors in 97L/STC1-treated group was found to be significantly smaller than the 97L/pLenti-treated group (*p*<0.05, Fig 4A). On the day of harvest (day 47), the tumors in the 97L/STC1-treated group were about 70% smaller than those in the 97L/pLenti-treated group (*p*<0.005, Fig 4A). A representative image is shown in Fig 4B. In parallel, the average tumor weight from the 97L/STC1-treated group was ~70% smaller than that in the 97L/pLenti-treated group (*p*< 0.05); the representative tumor masses are shown in Fig 4C, (*top*). We attempted to delineate the possible factors that might be involved in STC1-mediated reduction in tumor size. Total RNAs were extracted from the xenografts and pro-apoptotic genes related to inflammation such as interleukin-12 (*IL12A* and *B*) and NOD-like receptor family, pyrin domain containing 3(*NLRP3*) were measured using qPCR,. *IL12A*, *IL12B*, and *NLRP3* were found to be upregulated by 1.75-, 2.68- and 3.81-fold, respectively, in 97L/STC1 xenografts as compared with the 97L/pLenti xenografts (*p<0.05, **p<0.0001, Fig 4D).

## Discussion

Current evidence suggests that STC1 plays roles in inflammation and carcinogenesis, but the underlying mechanistic actions of STC1 remains unclear. Since HCC is an exemplified model of inflammation-related cancer, it represents a paradigm of studying the association between STC1 and tumor development. In the present study, *STC1* was found to be highly expressed in tumor tissues versus the adjacent normal counterpart, collected from the HCC cases. The expression of *STC1* was found to be positively associated with the levels of *IL6* and *IL8*. The greater expression levels of STC1 in tumor tissues have been reported in other types of cancers [7, 33, 38, 39], however the functional correlation between STC1, IL6 and IL8 expression is not clear. Experimental studies reported that STC1 regulates endothelial function via inhibiting function of tumor necrotic factor (TNF)-α in cardiovascular inflammation [40] and counteracted lipopolysaccharide (LPS)-induced inflammatory cascade in lungs [41]. Anti-inflammatory action of STC1 on endothelial cells and macrophages was reported [29] and was demonstrated in a model of anti-glomerular basement membrane disease in STC1-transgenic mice [32]. On the other hand, IL6 and IL8 are pro-inflammatory cytokines associated with high inflammatory reaction in HCC while the elevated secretion of IL6 and IL8 in HCC is known to promote metastasis and correlates with poor prognostic outcomes [42–46]. Intriguingly the STC1-High cohort was found to be an independent prognostic factor for overall survival among 216 HCC cases (S1 File). The observation is dissimilar to other type of cancer cases (i.e. human esophageal squamous cell carcinoma, colorectal carcinoma, and leukemia), in which STC1 was recognized as an unfavorable prognostic factor for post-operative outcome in patients [47–49]. Therefore, it is interesting to determine the roles of STC1 on the effects of pro-inflammatory cytokine on carcinogenesis of HCC.

With respect to tumor size analysis using the clinical data, the cohort of STC1-High showed smaller size as compared to the STC1-low group, but this was found only in large tumors (>6.3cm in diameter). This observation is agreeably to the fact that STC1 expression was induced in hypoxia, which occurs in large solid tumors [14]. STC1 is a target gene of hypoxia-inducible factor, which via gene transactivation by HIF-1α and/or IL-6-signaling [13, 14, 28]. Although the greater expression levels of STC1 in tumor samples could be interpreted as its pro-oncogenic role in tumor progression, the counteracting effects STC1 on the pro-inflammatory effects of IL6 and IL8 might slow down the process of carcinogenesis. This assumption is supported by our in vitro HCC cell-line studies in which STC1 inhibited the pro-migratory effects of IL6 and IL8, and reduced sizes of tumor spheroids. Moreover, the xenograft animal model in which STC1-overexpressing 97L cells grew slower than the control cells further reinforces this notion. Nonetheless, the underlying mechanism on how STC-1 reduced tumor growth has not been revealed in this study. In the fact that growth-related function of STC1 was suggested in related to the pro- or anti-apoptotic effects of STC1 [7]. The anti-apoptotic effect of STC1 was reported in different cell models, including thapsigargin-treated Paju cells [50], hypoxia-induced lung cancer cells [51], and in human ovarian cancer cells [9]. Yet the pro-apoptotic effects of STC1 were demonstrated in cultured chondrocytes [52], oxidative stress-induced human nasopharyngeal cancer cells [15], trichostatin A treated human colorectal cancer cells [53] and in oxidative stressed mouse embryo fibroblasts [54]. Therefore both the anti-inflammatory and pro-apoptotic action of STC1 might reduce the growth of HCC cells in vitro and in vivo. To address the possible mechanisms of STC1-driven tumor suppression, the expression of some inflammatory and pro-apoptotic genes in 97L/STC1- and 97L/pLenti-derived tumor xenografts was measured and compared. We found that *IL12* (isoforms A and B) and *NLRP3* were upregulated in 97L/STC1-derived tumor xenografts. Both IL-12 and NLRP3 were reported to show antitumor effects on HCC progression [55, 56], and IL-12 was shown to induce the production of cytokines to promote apoptosis [55]. The NLRP3 is the most characterized inflammasome, acting as a danger signal sensor to initiate inflammation through recruitment of caspases [56]. The pro-apoptotic effect of STC1 was further noted in the condition of the HCC patients classified with both STC1-High and IL-6-High/IL-8-High (S1 Table). Particularly, a significant reduction in tumor sizes was shown in STC1/IL-8-High tumor (31% reduction versus STC1/IL-8-Normal, *p* = 0.025), but not in STC1/IL-6-High cases (22% reduction versus STC1/IL-6-Normal, *p* = 0.245), which may due to the small sample size of the STC1/IL-6-High cohort. Therefore, it warrants conducting a further large-scale study using well-characterized HCC patient samples with high expression levels of STC1, IL-6 and IL-8. Collectively, our data are the first to demonstrate that the suppressive effect of STC1 on HCC tumors is associated with the inflammatory milieu.

## Supporting Information

S1 Fig An overall survival curves according to STC1 gene expression levels. Overall survival curve for high (*green*) and normal/low (*blue*) expression of STC1 in HCC patients (n = 216) by Kaplan-Meier analysis. The cohort with STC1-High was not correlated to prolong survival in HCC.
(PDF)

S1 File STC1 overexpression reduced cell viability in HCC cells. Stable STC1 overexpression was verified in Hep3B cell line using V5 antibody (Figure A). Cell viability of Hep3B/STC1 cells was found to be significantly reduced upon 3 days of incubation using MTT proliferation assay (Figure B).
(PDF)

S2 File ARRIVE guidelines checklist. (DOC)

S1 Table Clinicopathological data on tumor size among HCC samples with different expression levels of STC1, IL6 and IL8. *p*<0.05 was considered as significant.
(PDF)
