# Supplementary material for: Stanniocalcin-1 Reduces Tumor Size in Human Hepatocellular Carcinoma
Source: PLoS One. 2015 Oct 15;10(10):e0139977. doi: 10.1371/journal.pone.0139977 (PMC4607425; doi:10.1371/journal.pone.0139977)
Supplement: S1 Fig — Overall survival curve for high (green) and normal/low (blue) expression of STC1 in HCC patients (n = 216) by Kaplan-Meier analysis. The cohort with STC1-High was not correlated to prolong survival in HCC. (PDF) [file pone.0139977.s001.pdf]

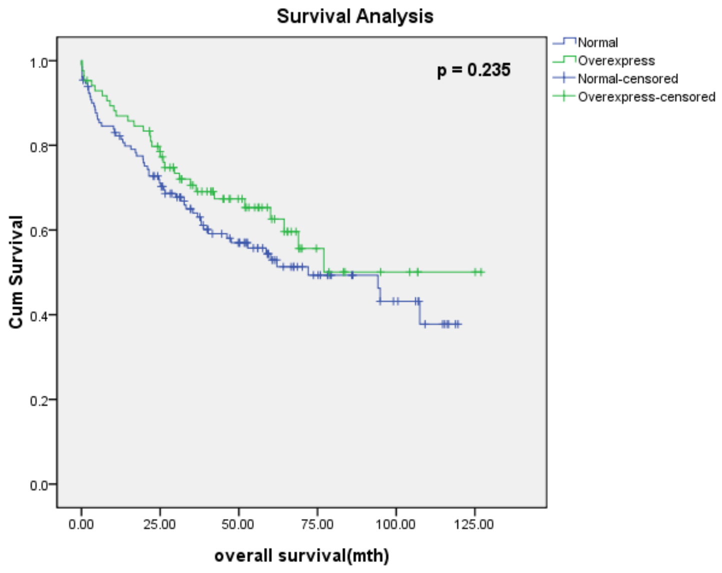

S1 Figure. Overall survival curves according to the STC1 gene expression levels. Overall survival curve for high (*green*) and normal/low (*blue*) expression of STC1 in HCC patients (n=216) by Kaplan-Meier analysis. The cohort with STC1-High was not correlated with prolong survival in HCC.
