# Supplementary material for: Stanniocalcin-1 Reduces Tumor Size in Human Hepatocellular Carcinoma
Source: PLoS One. 2015 Oct 15;10(10):e0139977. doi: 10.1371/journal.pone.0139977 (PMC4607425; doi:10.1371/journal.pone.0139977)
Supplement: S1 File — Stable STC1 overexpression was verified in Hep3B cell line using V5 antibody (Figure A). Cell viability of Hep3B/STC1 cells was found to be significantly reduced upon 3 days of incubation using MTT proliferation assay (Figure B). (PDF) [file pone.0139977.s002.pdf]

A

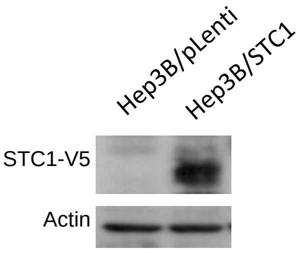

B

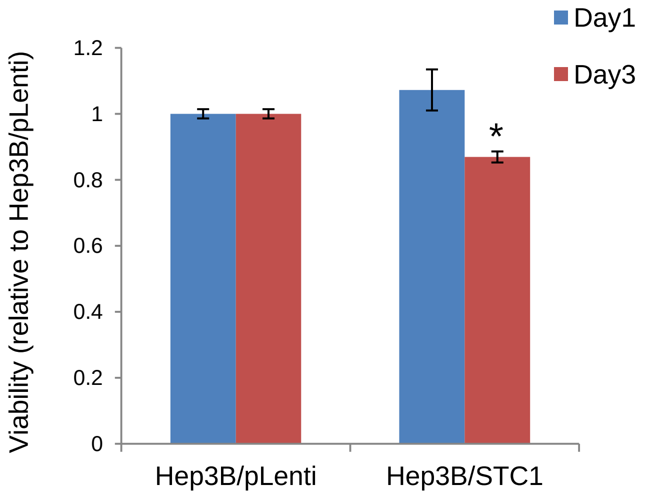

S1 File. STC1 overexpression reduced cell viability in HCC cells. Stable STC1 overexpression was verified in Hep3B cell line using V5 antibody (Figure A). Cell viability of Hep3B/STC1 cells was found to be significantly reduced upon 3 days of incubation using MTT proliferation assay (Figure B).
