# Supplementary material for: Stanniocalcin-1 Reduces Tumor Size in Human Hepatocellular Carcinoma
Source: PLoS One. 2015 Oct 15;10(10):e0139977. doi: 10.1371/journal.pone.0139977 (PMC4607425; doi:10.1371/journal.pone.0139977)
Supplement: S2 File — (DOC) [file pone.0139977.s003.doc]

**The ARRIVE Checklist**

**TITLE**

**1 Provide as accurate and concise a description of the content of the article as possible.**

Stanniocalcin-1 Reduces Tumor Size in Human Hepatocellular Carcinoma

**ABSTRACT**

**2 Provide an accurate summary of the background, research objectives (including details of the species or strain of animal used), key methods, principal findings, and conclusions of the study.**

Growing evidence has revealed high expression levels of stanniocalcin-1 (STC1) in different types of human cancers. Numerous experimental studies using cancer cell lines have demonstrated that STC1 is involved in inflammatory and apoptotic responses, however the role of STC1 in carcinogens is remains elusive. With hindsight, we conducted a statistical analysis of the expression levels of STC1 and clinico-pathological data from216 patients with hepatocellular carcinoma (HCC). We found that STC1 was upregulated in tumor tissues and positively correlated with the levels of interleukin (IL)-6 and IL-8. Intriguingly tumors with higher levels of STC1 expression (tumor/normal ≥ 2) were significantly smaller than those with lower levels (tumor/normal<2) (p=0.008). A pharmacological approach was adopted to reveal the functional correlation between STC1 and the ILs in the HCC cell-line Hep3B. STC1 expression was induced by IL-6 and IL-8; however, this induction suppressed the pro-migratory effects of IL-6 and IL-8. Overexpression of STC1 reduced size of the tumor spheroids. The suppressive effect of STC1 on tumor growth was confirmed in vivo using stably STC1-overexpressing 97L cells in a mouse xenograft model. Genetic analysis showed upregulation of the pro-apoptotic genesinterleukin-12(IL12) and NOD-like receptor family, pyrin domain-containing 3(NLRP3) associated with the inhibition of tumor cell growth. This study suggests that STC1 is a potential therapeutic target for inflammatory tumors in patients with HCC.

**INTRODUCTION**

**Background**

**3 a. Include sufficient scientific background (including relevant references to previous work) to understand the motivation and context for the study, and explain the experimental approach and rationale.**

**b. Explain how and why the animal species and model being used can address the scientific objectives and, where appropriate, the study’s relevance to human biology.**

1. We selected the mouse model because it is a common strain that used for xenograft model for studying tumor growth *in vivo* based on severe defects characteristic in innate and adaptive immunity.
2. This model helped us to understand the effect of STC1 in hepatpcarinoma progression *in vivo* and the result could potentially aid distinguishing a therapeutic target for hepatocarcinoma.

**Objectives**

**4 Clearly describe the primary and any secondary objectives of the study, or specific hypotheses being tested.**

1, To determine the expression of STC1 in human hepatocarinoma.

2, To verify the role of STC1 in hepatocarinoma *in vivo* .

**METHODS**

**Ethical statement**

**5 Indicate the nature of the ethical review permissions, relevant licenses (e.g. Animal [Scientific Procedures] Act 1986), and national or institutional guidelines for the care and use of animals, that cover the research.**

Animal experiments were approved by the Animals (Control of Experiments) Ordinance 340, Hong Kong, and were conducted according to the guidelines of the Committee on the Use of Live Animals in Teaching and Research (CULATR), The University of Hong Kong, Hong Kong (Ref: 2825-12).

**Study design**

**6 For each experiment, give brief details of the study design, including:**

**a. The number of experimental and control groups.**

**b. Any steps taken to minimise the effects of subjective bias when allocating animals to treatment (e.g., randomisation procedure) and when assessing results (e.g., if done, describe who was blinded and when).**

**c. The experimental unit (e.g. a single animal, group, or cage of animals).**

**A time-line diagram or flow chart can be useful to illustrate how complex study designs were carried out.**

a. Ten of 6–7 week-old male mice (BALB-c nude) were used for the experimental and control groups.

b-c. Two of independent experiment were done with different lot of animals.

**Experimental procedures**

**7 For each experiment and each experimental group, including controls, provide precise details of all procedures carried out. For example:**

**a. How (e.g., drug formulation and dose, site and route of administration, anaesthesia and analgesia used [including monitoring], surgical procedure, method of euthanasia). Provide details of any specialist equipment used, including supplier(s).**

**b. When (e.g., time of day).**

**c. Where (e.g., home cage, laboratory, water maze).**

**d. Why (e.g., rationale for choice of specific anaesthetic, route of administration, drug dose used).**

1. All mice were anaesthetic by Pentobarbital and then sacrificed by cervical dislocation. Mouse images were captured.
2. 2:00pm
3. Laboratory Animal Unit of The University of Hong Kong
4. Pentobarbital (50mg/kg, i.p.) is commonly used.

**Experimental animals**

**8 a. Provide details of the animals used, including species, strain, sex, developmental stage (e.g., mean or median age plus age range), and weight (e.g., mean or median weight plus weight range).**

**b. Provide further relevant information such as the source of animals, international strain nomenclature, genetic modification status (e.g. knock-out or transgenic), genotype, health/immune status, drug- or test naıve, previous procedures, etc.**

1. Wild-type nude mice (BALB-c nude), 6-8 weeks, male, 18-20 g
2. All animals are bred in Laboratory Animal Unit of The University of Hong Kong.

**Housing and husbandry**

**9 Provide details of:**

**a. Housing (e.g., type of facility, e.g., specific pathogen free (SPF); type of cage or housing; bedding material; number of cage companions; tank shape and material etc. for fish).**

**b. Husbandry conditions (e.g., breeding programme, light/dark cycle, temperature, quality of water etc. for fish, type of food, access to food and water, environmental enrichment).**

**c. Welfare-related assessments and interventions that were carried out before, during, or after the experiment.**

a-b: Mice were housed in individually ventilated cages (IVC, up to six mice each cage) under constant environmental condition with a 12-hour light/12-hour dark cycle, and with free access to autoclaved water and chow.

c. Not applicable

**Sample size**

**10 a. Specify the total number of animals used in each experiment and the number of animals in each experimental group.**

**b. Explain how the number of animals was decided. Provide details of any sample size calculation used.**

**c. Indicate the number of independent replications of each experiment, if relevant.**

For each set, 10 mice were used for each experimental and control groups. Two independent sets of experiments were done to control the lot variations.

**Allocating animals to experimental groups**

**11 a. Give full details of how animals were allocated to experimental groups, including randomisation or matching if done.**

**b. Describe the order in which the animals in the different experimental groups were treated and assessed.**

Mice were randomly divided into an experimental or a control group.

**Experimental outcomes**

**12 Clearly define the primary and secondary experimental outcomes assessed (e.g., cell death, molecular markers, behavioural changes).**

The volume of tumor was continually measured until reaching approximately 700 mm^3^.

**Statistical methods**

**13 a. Provide details of the statistical methods used for each analysis.**

**b. Specify the unit of analysis for each dataset (e.g. single animal, group of animals, single neuron).**

**c. Describe any methods used to assess whether the data met the assumptions of the statistical approach.**

The data were analyzed by a Student’s t-test or one-way analysis of variance (ANOVA) followed by Duncan’s multiple range test. All data are represented as statistical means ± SEM. A p-value of < 0.05 was considered statistically significant.

**RESULTS**

**Baseline data
14 For each experimental group, report relevant characteristics and health status of animals (e.g., weight, microbiological status, and drug- or test-naıve) before treatment or testing (this information can often be tabulated).**

All animals analyzed were in good health.

**Numbers analysed**

**15 a. Report the number of animals in each group included in each analysis. Report absolute numbers (e.g. 10/20, not 50%).**

**b. If any animals or data were not included in the analysis, explain why.**

10/10 for both experimental and control groups.

**Outcomes and estimation**

**16 Report the results for each analysis carried out, with a measure of precision (e.g., standard error or confidence interval).**

All data are represented as statistical means ± SEM.

**Adverse events**

**17 a. Give details of all important adverse events in each experimental group.**

**b. Describe any modifications to the experimental protocols made to reduce adverse events.**

There were no adverse events.

**DISCUSSION**

**Interpretation/scientific implications**

**18 a. Interpret the results, taking into account the study objectives and hypotheses, current theory, and other relevant studies in the literature.**

**b. Comment on the study limitations including any potential sources of bias, any limitations of the animal model, and the imprecision associated with the results.**

**c. Describe any implications of your experimental methods or findings for the replacement, refinement, or reduction (the 3Rs) of the use of animals in research.**

There might be cell line differences in the response of xenograft growth, but may not affect the general trend of the result.

**Generalisability/translation**

**19 Comment on whether, and how, the findings of this study are likely to translate to other species or systems, including any relevance to human biology.**

This study does not imply that human heptocarcinoma will be identical with mouse xenograft development. However, it gives us a shed light for the effect of STC1 in tumor microenvironment and supports the finding from *in vitro* assay.

**Funding**

**20 List all funding sources (including grant number) and the role of the funder(s) in the study.**

This work was supported by the General Research Fund (HKBU 261610), University Grants Committee (CKC Wong).
