# Supplementary material for: Stanniocalcin-1 Reduces Tumor Size in Human Hepatocellular Carcinoma
Source: PLoS One. 2015 Oct 15;10(10):e0139977. doi: 10.1371/journal.pone.0139977 (PMC4607425; doi:10.1371/journal.pone.0139977)
Supplement: S1 Table — p<0.05 was considered as significant. (PDF) [file pone.0139977.s004.pdf]

		Tumor size (cm)		
	Frequency	mean	SD	p -value
IL6-Normal	199	7.29	4.24	0.528
IL6-High	17	6.77	3.11	
IL8-Normal	185	7.34	4.18	0.391
IL8-High	31	6.67	4.03	
STC1-Normal	131	7.841	4.32	**0.008**
STC1-High	85	6.359	3.75	
STC1/IL6 -Normal	207	7.313	4.21	0.245
STC1/IL6 -High	9	5.667	2.11	
STC1/IL8 -Normal	202	7.4	4.2	**0.025**
STC1/IL8 -High	14	5.1	3.2	

* Incomplete patient information

S1 Table. Clinicopathological data on tumor size among HCC samples with different expression levels of STC1, IL6 and IL8. p<0.05 was considered as significant.
